# Autocrine Transforming  Growth Factor-β Growth Pathway in Murine Osteosarcoma Cell Lines Associated with Inability to Affect Phosphorylation of Retinoblastoma Protein

**DOI:** 10.1080/13577140020008057

**Published:** 2000-09

**Authors:** Fariba Navid, John J. Letterio, Choh L. Yeung, Michiel Pegtel, Lee J. Helman

**Affiliations:** 1 Pediatric Oncology Branch National Cancer Institute National Institutes of Health Bethesda MD 20892-1928 USA; 2 Laboratory of Cell Regulation and Carcinogenesis National Cancer Institute National Institutes of Health Bethesda MD 20892-1928 USA

## Abstract

*Purpose.* Production of active transforming growth factor-β (TGF-β ) by human osteosarcoma may contribute to malignant progression through mechanisms that include induction of angiogenesis, immune suppression and autocrine growth stimulation of tumor cell growth.To study events associated with induction of cell proliferation by TGF-β , we have evaluated the TGF-β pathway in two murine osteosarcoma cell lines, K7 and K12.

*Results.* Northern and immunohistochemical analyses show that each cell line expressesTGF-β1 and TGF-β3 mRNA and protein. Both cell lines secrete activeTGF-β 1 and display a 30–50% reduction in growth when cultured in the presence of a TGF-β blocking antibody. Expression of TGF-β receptors TβRI, TβRII and TβRIII is demonstrated by affinity labeling with ^125^ -TGF-β 1, and the intermediates, Smads 2, 3 and 4, are uniformly expressed. Smads 2 and 3 are phosphorylated in response toTGF-β , while pRb phosphorylation in each osteosarcoma cell line is not affected by either exogenousTGF-β or TGF-β antibody.

*Conclusions.* The data implicate events downstream of Smad activation, including impaired regulation of pRb, in the lack of a growth inhibitory response toTGF-β , and indicate that this murine model of osteosarcoma is valid for investigating the roles of autocrineTGF-β *in vivo.*


Sarcoma (2000) 4, 93–102

ORIGINAL ARTICLE

### Autocrine transforming growth factor-β growth pathway in murine osteosarcoma cell lines associated with inability to affect phosphorylation of retinoblastoma protein

FARIBA NAVID,[1] JOHN J. LETTERIO,[2] CHOH L. YEUNG,[1] MICHIEL PEGTEL[2] & LEE J. HELMAN[1]

[1]*Pediatric Oncology Branch and* [2]*Laboratory of Cell Regulation and Carcinogenesis, National Cancer Institute, National Institutes of Health, Bethesda, MD 20892-1928, USA*

**Abstract**

*Purpose*. Production of active transforming growth factor-β (TGF-β) by human osteosarcoma may contribute to malignant progression through mechanisms that include induction of angiogenesis, immune suppression and autocrine growth stimulation of tumor cell growth. To study events associated with induction of cell proliferation by TGF-β, we have evaluated the TGF-β pathway in two murine osteosarcoma cell lines, K7 and K12.

*Results*. Northern and immunohistochemical analyses show that each cell line expresses TGF-β1 and TGF-β3 mRNA and protein. Both cell lines secrete active TGF-β1 and display a 30–50% reduction in growth when cultured in the presence of a TGF-β blocking antibody. Expression of TGF-β receptors TβRI, TβRII and TβRIII is demonstrated by affinity labeling with $^{125}$I-TGF-β1, and the intermediates, Smads 2, 3 and 4, are uniformly expressed. Smads 2 and 3 are phosphorylated in response to TGF-β, while pRb phosphorylation in each osteosarcoma cell line is not affected by either exogenous TGF-β or TGF-β antibody.

*Conclusions*. The data implicate events downstream of Smad activation, including impaired regulation of pRb, in the lack of a growth inhibitory response to TGF-β, and indicate that this murine model of osteosarcoma is valid for investigating the roles of autocrine TGF-β *in vivo*.

**Key words:** *osteosarcoma, transforming growth factor-β, Smad proteins, retinoblastoma protein*

### Introduction

Transforming growth factor-β (TGF-β) is a pleiotropic cytokine that is highly abundant in bone and is involved in various aspects of bone cell biology including replication, differentiation, osteogenesis and resorption.[1] Both stimulatory and inhibitory effects of TGF-β on the growth of cell lines derived from normal osteoblasts and osteosarcoma cells have been reported in the literature.[2–6] The mechanisms responsible for the variable effects of TGF-β on growth of osteoblastic cell lines are not clear. The recent reports suggesting a correlation between severity of disease and TGF-β expression in osteosarcoma, as assessed by immunohistochemistry in tissue samples, support the hypothesis that TGF-β plays a role in the pathogenesis of this malignant bone tumor.[7–9] An understanding of the mechanisms mediating the effects of TGF-β on the growth of osteosarcoma cells may give insight into the biology of this tumor.

Three mammalian isoforms of TGF-β (TGF-β1, -β2 and -β3) have been identified with nearly identical biological activity *in vitro*, and overlapping patterns of expression *in vivo*. TGF-β1 is widely expressed in most tissues throughout development and in the adult organism, while TGF-β3 is most strongly expressed in tissues of mesenchymal origin. Though they share overlapping activities in most culture systems, TGF-β3 is 3- to 10-fold more potent on a molar basis than TGF-β1 or TGF-β2 in fetal rat bone and in rat osteosarcoma cultures.[1,10]

The three isoforms signal through the same receptor complex but with different binding affinities.[11–13] Three major classes of receptors have been identified for TGF-β, namely a type I and type II receptor (TβRI and TβRII) and betaglycan (TβRIII). The type III receptor is a large cell surface proteoglycan whose function is unclear, though it may be involved in the presentation of ligand to the type II receptor.[14,15] The TβRI and TβRII are transmembrane serine/

Correspondence to: F. Navid, MD, Pediatric Oncology Branch, National Cancer Institute, National Institutes of Health, 10 Center Drive, MSC-1928, Building 10, Room 13N240, Bethesda, MD 20892-1928, USA. Tel: 301 496-5506; Fax: 301 402-0575; E-mail: navidf@mail.nih.gov

1357-714Xprint/1369-1643online/00/030093-10 © 2000 Taylor & Francis Ltd

*DOI: 10.1080/13577140020008057*

threonine kinases. Upon ligand binding, the type II receptor phosphorylates the type I receptor, which mediates downstream signaling through the Smad family of proteins.[16] The pathway-restricted Smad2 and Smad3 proteins are phosphorylated by the type I receptor and then form a complex in the cytoplasm with Smad4, a signaling component common to many TGF-β family proteins. This complex translocates to the nucleus and mediates activation of target genes.[17–21] The convergence of the TGF-β pathway with vitamin D signaling pathways through Smad transcriptional activators implicates their importance in modulating bone mineralization and bone growth.[22,23]

TGF-β appears to chiefly block progression through the mid/late G1 phase of the cell cycle by affecting expression and function of a number of cell cycle regulatory proteins.[24,25] TGF-β has been shown to either downregulate expression or decrease the activity of cdk2, cdk4 and cyclin E. In addition, TGF-β treatment induces the expression and functional activity of the cyclin-dependent kinase inhibitors (CDKIs). $p15^{INK4B}$ and $p21^{Cip1}$ are upregulated in a variety of cell types, whereas the distribution of $p27^{Kip1}$ is altered in response to TGF-β.[26–28] These alterations in cdks, cyclins and CDKIs block entry into the S phase of the cell cycle by directly and indirectly preventing the phosphorylation of the retinoblastoma protein (pRb).[29–31]

The TGF-β ligands and signaling intermediates play complex roles in tumorigenesis. Altered expression and mutational inactivation of the TGF-β receptors and downstream effectors, including Smad2 and Smad4, have been shown to prevent the growth inhibitory effects of TGF-β and contribute to enhanced tumorigenesis.[32–36] However, the role of TGF-β ligands in carcinogenesis appears to be more complex, and is in part attributed to the pleiotropic activity of TGF-β, including the ability to act as an autocrine, paracrine and sometimes endocrine growth factor. TGF-β has been shown to be involved in tumor invasion and metastasis, stromal matrix formation, immunosuppression and angiogenesis.[37–40] Tumor cells that become resistant to the growth inhibitory effects of TGF-β often secrete an active form of the protein, which acts in a paracrine fashion to promote tumor progression by virtue of such effects on surrounding tissues.[41] Indeed, in murine models of carcinogenesis involving TGF-β1 heterozygote knock-out mice, loss of heterozygosity at the TGF-β1 locus does not occur within tumor tissue, presumably due to the selective allelic retention and probable promoting effects of TGF-β1 in the tumor microenvironment.[42]

In this report, we describe the patterns of TGF-β expression and the response to this cytokine in two clonal murine osteosarcoma cell lines, K7 and K12, derived from a single spontaneously occurring tumor in a BALB/c mouse. These two cell lines exhibit distinct morphologic and biologic differences. The histology as well as the biology of these cell lines injected into immunocompetent mice mimic those of human osteosarcoma. The tumors are aggressive, produce osteoid, express bone markers and have varying metastatic potential.[43] Using this murine model for human osteosarcoma, we examine components of the TGF-β pathway frequently altered in human tumors, including the receptors, Smad proteins and the retinoblastoma protein. The results show that TGF-β acts as an autocrine growth factor in this murine model of osteosarcoma, stimulating proliferation in both K7 and K12 cell lines. The data also represent the first evidence for normal ligand-induced phosphorylation of Smad2 and Smad3 in osteosarcoma, and show that the disruption of these events does not underlie the altered regulation of retinoblastoma protein phosporylation in osteosarcoma.

### Materials and methods

### *Cell lines*

K7 and K12 are two clonal cell lines that were established and characterized by Schmidt *et al.* from a single spontaneously occurring murine osteosarcoma.[43] Cell lines were grown in Dulbecco's modified Eagle's medium (DMEM) supplemented with 10% fetal bovine serum (FBS), 100 U/ml penicillin, 100 μg/ml streptomycin and 2 mM L-glutamine at 37°C and 5–6% $CO_2$. The TGF-β sensitive Mv1Lu (CCL64) cells were maintained subconfluent in DMEM (high glucose) containing L-glutamine, 10% FBS and 1% penicillin/streptomycin.

### *TGF-β assays*

*Conditioned media preparation.* A total of $3\times10^6$ osteosarcoma cells (K7 and K12) were cultured for up to 48 h in 2 ml of serum-free medium, supplemented with 5 ml ITS+ (insulin, transferrin, selenium) culture supplement (Collaborative Biomedical, Bedford, MA, lot no. 901837). Culture supernatants were collected, centrifuged at 10 000×*g* at 4°C to pellet any cells or debris, and supernatant was transferred to siliconized tubes with protease inhibitors (leupeptin, pepstatin and aprotinin, 1 mg/ml) and stored at −70°C.

*TGF-β bioassay.* A modified Mv1Lu bioassay was used. Cells were plated at $1\times10^4$ cells/well in 96 well plates with 200 μl of DMEM containing 10% FBS, and incubated for 8–12 h at 37°C to ensure complete adherence. Media was aspirated and replaced with serial dilutions of each conditioned media (diluted in DMEM with 0.5% FBS), in the presence or absence of 30 μg/ml of either the pan-specific mouse monoclonal anti-TGF-β blocking antibody 1D11 (Genzyme, Cambridge, MA) or control IgG; additional wells were treated with media plus TGF-β

standard concentrations in a total volume of 150 μl/well (each condition was performed in triplicate). Plates were subsequently incubated for an additional 24 h, and 1 mCi of [$^3$H]thymidine was added for the final 2 h of the incubation. Media was aspirated and replaced with 50 μl of Trypsin-ethylenediaminetetraacetic acid (EDTA)/well, and cells were incubated for 30 minutes at 37°C prior to harvesting onto 96-well filter plates which were processed with a Top Count Microplate Scintillation Reader according to manufacturer's instructions (Packard Instrument Company, Meriden, CT).

*TGF-β enzyme-linked immunosorbent assay (ELISA) assay.* Performed using an TGF-β1 immunoassay kit (R&D Systems, Minneapolis, MN).[44]

### *Immunohistochemical detection of TGF-β isoforms in tumor tissue*

Tumor tissue was fixed in neutral buffered formalin and embedded in paraffin. Sections 5 μM thick were stained with hematoxylin and eosin for routine histology. Additional sections were evaluated with isoform-specific anti-TGF-β antibodies directed against TGF-β1, TGF-β2 and TGF-β3, followed by peroxidase staining as previously described.[45–47]

### *TGF-β blocking studies with antibody and latency-associated peptide*

K7 and K12 cell lines were plated in triplicate in 96 well plates in DMEM with 10% FBS at a density of $5\times10^3$/well and $7.5\times10^3$/well, respectively. Twenty-four hours later, cells were washed twice with 1XPBS (phosphate buffered saline without calcium and magnesium, pH 7.4) and incubated with pan-specific anti-TGF-β antibody or recombinant TGF-β1 latency-associated peptide (LAP) in 200 μl of 0.5% FBS, DMEM and 2 μl of ITS+premix containing insulin, transferrin, selenious acid, bovine serum albumin (BSA) and linoleic acid (Beckton Dickinson Labware, Bedford, MA); 10 μg/ml class-matched mouse IgG1, k (MOPC-21) (Sigma BioSciences, St Louis, MO), was used as control. Media was changed every 48 h. Cells were harvested at 48, 72 and 96 h after treatment. Cell number was determined using colorimetric MTT (3-(4,5-diMethylThiazol-2-yl)-2,5-diphenyl Tetrazolium bromide) (Sigma Chemical Co., St Louis, MO) assay as previously described.[48,49] Absorbencies were measured on each test well on a Titertek enzyme-linked immunosorbent assay reader using a test wavelength of 570 nm and a reference wavelength of 690 nm. The optical density measurements were converted to cell number using a standard curve.

### *Studies with exogenous TGF-β1*

K7 and K12 cell lines were plated in triplicate in 96 well plates in DMEM with 10% FBS at a density of $5\times10^3$/well and $7.5\times10^3$/well, respectively. Twenty-four hours later, cells were washed twice with 1XPBS and incubated with 10 ng/ml of TGF-β1 in 200 μl of serum-free DMEM, and ITS+premix. The control cells were treated with HB buffer (4 mM HCl and 1 mg/ml BSA). HB buffer is the vehicle for the exogenous TGF-β. Cells were harvested at 24 and 48 h after treatment. Cell number was determined using MTT assay as described above.

### *Western blot analysis*

Cells were grown to approximately 50–60% confluency. The cells were washed twice with 1XPBS and switched to serum-free media. After 24 h the media was changed to 5% FBS and DMEM plus the designated treatment (10 ng/ml TGF-β1, 10 ng/ml TGF-β3, HB buffer, MOPC, 5 μg/ml antibody to TGF-β or no treatment). Cells were harvested after 24 h of incubation. A standard Western blotting procedure as described by Pharmingen (San Diego, CA) was used except that the primary antibody was diluted in 1% gelatin in PBS, 0.1% azide and the secondary antibody was diluted in 1% gelatin in PBS. The specific antibodies used were as follows: purified mouse anti-human Rb antibody protein (catalog no. 14001A, Pharmingen, San Diego, CA), mouse monoclonal anti-Smad4 (B-8) (Santa Cruz Biotechnology, Inc., Santa Cruz, CA) and anti-mouse horseradish peroxidase (HRP) conjugated IgG (light and heavy chain) polyclonal antibody (Santa Cruz Biotechnology, Inc., Santa Cruz, CA). The concentration of protein for each sample was determined using Bio-Rad DC Protein Assay (BIO-RAD Laboratories, Hercules, CA).

### *TGF-β receptor cross-linking*

Osteosarcoma cells were grown to confluence in six well plates, washed three times in ice cold binding buffer (DMEM high glucose, 25 mM Hepes, pH 7.4, 1 mg/ml BSA), and incubated in 2 ml of the same buffer plus $^{125}$I-TGF-β1 (0.35 μCi; Dupont/NEN, Boston, MA) with or without an excess of unlabeled TGF-β1 (120 ng) to compete for specific binding of radiolabeled ligand. Plates were incubated on a rocker at 4°C for 2.5 h, washed three times in cold wash buffer (DMEM, 25 mM Hepes, pH 7.4) and incubated in 1 ml of the same buffer containing 3 mM disuccinimidyl suberate (Pierce, Rockford, IL). After incubating for 1 h at 4°C, cells were washed three times in cold sucrose buffer (250 mM sucrose, 10 mM Tris, pH 7.4, 1 mM EDTA) and lysed in 200 μl of 1XRIPA (1XPBS, 1% NP40, 0.5% sodium deoxycholate, 0.1% SDS) with protease inhibitors (200 nM AEBSF, 1 μg/ml leupeptin, pepstatin and aprotinin) added immediately prior to cell lysis. Radiolabeled proteins were separated on 8% Tris-Glycine polyacrylamide gels (NOVEX, San Diego, CA). Gels were dried and exposed to Kodak Xomat film (Kodak, Rochester, NY).

*Smad immunoprecipitation*

Cells were grown to confluence in 100 mm tissue culture dishes, washed twice with phosphate-free DMEM and incubated with 2 ml phosphate-free DMEM and 0.75 mCi [$^{32}$P] orthophosphate at 37°C in an incubator for 90 minutes; 10 ng/ml of TGF-β1 and 10 ng/ml of TGF-β3 was added to the designated plate for each cell line and incubated for another 30 minutes. Cells were washed with cold PBS, counted, lysed with RIPA buffer (plus protease inhibitors) and incubated at 4°C for 30 minutes. Cells were then harvested with cell scrapers and spun at 12 000 r.p.m. at 4°C for 20 minutes. Supernatant was stored at –70°C until ready to use. Protein G (50 μl per reaction) (Santa Cruz Biotechnology, Inc., Santa Cruz, CA) with Smad2 (1 μg per reaction) mouse monoclonal antibody (catalog no. S66220, Transduction Laboratories, Lexington, NY) or MOPC-21 (1 μg per reaction) was incubated overnight at 4°C on a shaker; 50 μl of protein G plus the antibody was mixed with one-third of the protein lysates (exact amount was adjusted for cell count), and incubated with continuous shaking at 4°C. The immunoprecipitates were collected by centrifugation at 2500 r.p.m. for 5 minutes at 4°C. The pellet was washed four times with PBS, resuspended in Tris-Glycine SDS sample buffer (NOVEX, San Diego, CA), and boiled for 5 minutes. The agarose beads were pelleted by centrifugation and the supernatant was loaded on an 8% Tris-Glycine polyacrylamide gel. Electrophoresis was carried out at 125 V. The gel was dried and exposed to Kodak BioMax Light Film (Kodak, Rochester, NY).

### Results

*TGF-β is an autocrine growth factor in K7 and K12 cells*

Expression of both TGF-β1 and TGF-β3 mRNA was detected by Northern analysis (β1>β3) in both K7 and K12 cell lines, while there was no detectable expression of TGF-β2 (data not shown). Immunohistochemical studies of sections from tumors of K7 and K12 grown in a syngeneic mouse strain reveal abundant expression of both TGF-β1 and TGF-β3, with no detectable TGF-β2 protein (Fig. 1).

Culture of both K7 and K12 in serum-free conditions revealed the production of active TGF-β by both cell lines. Using an ELISA assay, we determined the level of TGF-β1 secreted into conditioned media over a 48 h period to be 8.38 ng/ml for K7 and 0.94 ng/ml for K12. TGF-β is secreted as a latent precursor by most normal cells, but is produced in an active state by a variety of tumors. Inhibition of the growth of mink lung epithelial (Mv1Lu) cells was used to confirm the presence of active TGF-β in conditioned media, and reveals that 40–50% of the TGF-β secreted by K7 and K12 is in the active form (Fig. 2).

We next sought to determine whether the endogenously secreted TGF-β had any autocrine biological activity. Growth of either K7 or K12 in the presence of 5 μg/ml or 10 μg/ml of a pan-specific blocking monoclonal antibody resulted in a 50% reduction in cell number when compared to their growth in the presence of an IgG control antibody. (Fig. 3A, B). Concentrations of antibody up to 30 μg/ml did not result in further growth inhibition (data not shown). To confirm this autocrine stimulatory effect of endogenous TGF-β on cell growth, K7 and K12 cells were treated with recombinant TGF-β1 LAP, a potent inhibitor of biologically active TGF-β.[50] Treatment with 250 ng/ml of LAP resulted in 38% and 32% decrease in growth of K7 and K12 cells respectively, when compared to PBS-treated control cultures (Fig. 3C, D).

*Mitogenic response to exogenous TGF-β in K7 and K12 cells*

Though both K7 and K12 cells secrete TGF-β, the effect of exogenous TGF-β on their growth was also examined. Exposure of K7 and K12 cell cultures grown in serum-free media to 10 ng/ml of TGF-β1 resulted in a greater than 60% increase in the rate of growth in each cell line, when compared to the solvent-treated control cultures (Fig. 4).

*TGF-β receptors*

Abnormalities in the expression of both TβRI and TβRII have been reported in primary human tumors, and in established tumor cell lines that are insensitive to growth inhibitory effects of TGF-β. To assess the receptor status in the K7 and K12 cell lines, an $^{125}$I labeled TGF-β1 affinity binding assay was performed. All three TGF-β receptors are present on the cell surface in each cell line, and appear at the expected size (Fig. 5). Interestingly, there is an additional band that migrates between the TβRII and TβRIII in both cell lines, though more prominent in K12 than in K7. Immunoprecipitation of this band with an antibody to betaglycan suggest that this band may represent an alternately glycosylated form of TβRIII (data not shown).

*Phosphorylation of Smad2 similar in Mv1Lu and osteosarcoma cell lines*

The established role of both Smad2 and Smad4 as tumor suppressor genes highlights the importance of their function in conveying the growth inhibitory signals from TGF-β family ligands. Their function in the mitogenic response to TGF-β in osteosarcoma is not known. We find no difference in the phosphorylation of Smad2 in response to TGF-β inhibition of Mv1Lu (CCL64) cell growth or the mitogenic response of either osteosarcoma cell line (Fig. 6). A smaller band just below Smad2 was detected in the TGF-β-treated cells in the [$^{32}$P] orthophosphate labeled immunoprecipitates with anti-Smad2. This

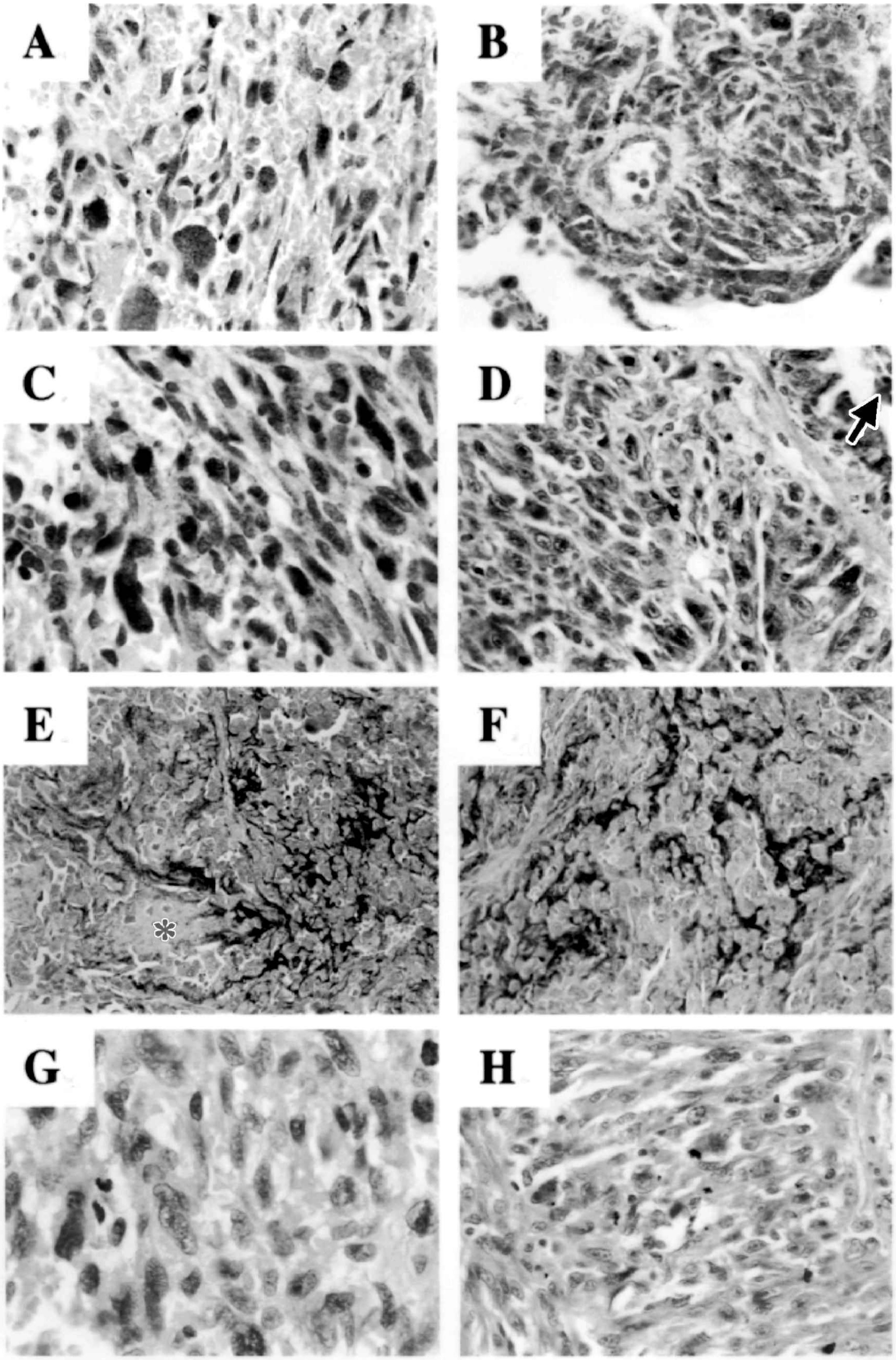

**Figure 1**. *Expression of TGF-β isoforms in murine K7 osteosarcomas, both in primary (A, C, E, G) and pulmonary (B, D, F, H) metastatic tissue. Immunostaining of sections with the LC antibody to TGF-β1 reveals intracellular localization (A, B), while staining with the CC antibody to TGF-β1 (E, F) generates the typical pattern of extracellular matrix-associated localization frequently observed with this antibody, primarily in areas where osteoid is also observed (asterisk in E). The anti-TGF-β3 antibody (C, D) also strongly reacts with tumor cells and can be seen in normal columnar epithelia of the lung for comparison (arrowhead in D). Immunostaining of sections with normal rabbit serum (G, H) are the negative controls. Comparable results were obtained for tumors derived from K12 cells.*

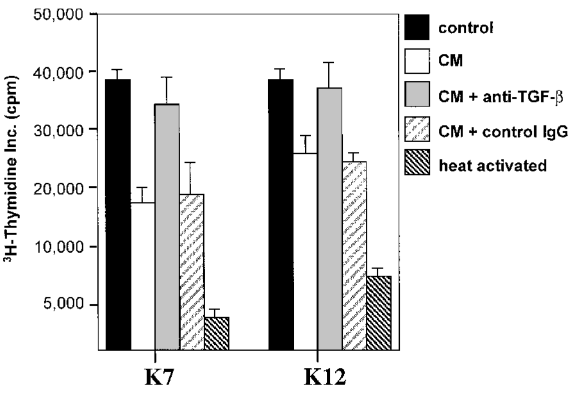

**Figure 2.** *Production of active TGF-β by K7 and K12 murine osteosarcomas was determined by a bioassay with the TGF-β-sensitive mink lung epithelial cell line CCL64. CCL64 cells were plated at subconfluence. Growth in normal media (black bars) was compared with growth in the presence of a 10-fold dilution of media conditioned by each osteosarcoma cell line (CM, white bars) as described in 'Materials and methods'. The addition of a pan-specific TGF-β blocking antibody to CM (gray bars) results in the reversal of inhibition of CCL64 cells by CM. The specificity of inhibition is indicated by the inability of control IgG to block the effects of CM. Treatment of CM at 80 °C for 8 minutes activates any latent TGF-β produced by the osteosarcoma cells, and leads to further inhibition of the growth of CCL64 cells.*

slightly smaller band is the appropriate size for Smad3, which is highly homologous with Smad2 and reacts with the antibody used for immunoprecipitation. Smad 4 was also detected in K7 and K12 cells, at levels similar to that found in CCL64 cells (Fig. 7).

### *Phosphorylation of pRb in response to TGF-β*

An important target in the growth arrest response to TGF-β in Mv1Lu cells is pRb, which is maintained in a dephosphorylated state following exposure to this cytokine. To assess the pattern of pRb phosphorylation in response to TGF-β in K7 and K12 cells, we cultured each in the presence of either exogenous TGF-β1 or a TGF-β blocking antibody. Neither treatment affects the phosphorylation state of pRb in either K7 or K12. However, as previously described, pRB is dephosphorylated in Mv1Lu in response to TGF-β (Fig. 8).

### Discussion

In the present study, we show that TGF-β acts as an autocrine stimulator of growth in two murine osteosarcoma cell lines, K7 and K12. Our experiments

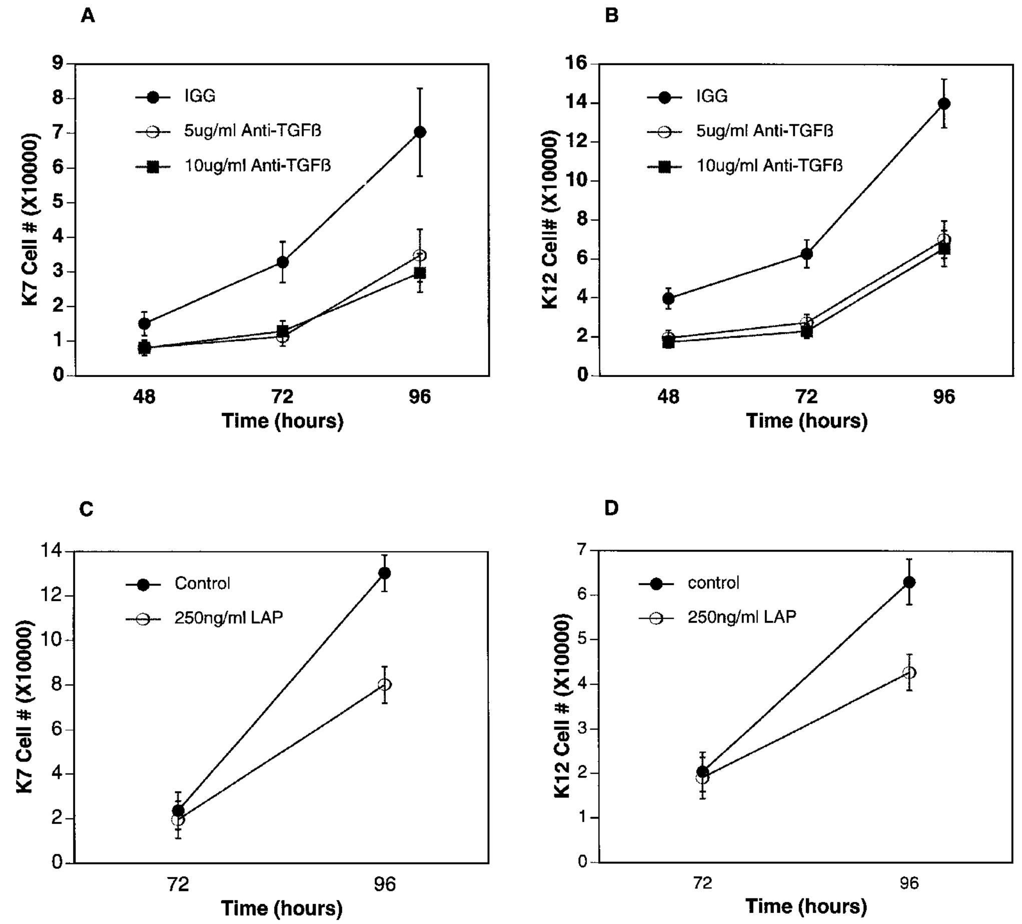

**Figure 3.** *Effect of pan-specific TGF-β monoclonal antibody (A and B) and LAP (C and D) on the growth of K7 and K12 osteosarcoma cell lines. K7 and K12 cells were plated in media containing 10% FBS at a density of 5×10³/well and 7.5×10³/well, respectively. Twenty-four hours later, the cells were incubated with 10 μg/ml or 5 μg/ml anti-TGF-β monoclonal mouse anti-TGF-β1,-β2, -β3 IgG1 or 250 ng/ml recombinant TGF-β1 LAP in media containing 0.5% FBS; 10 μg/ml class-matched mouse IgG1, k (MOPC-21), was used as control for the antibody and PBS was used as a control for LAP. Cells were harvested at 48, 72 and 96 h after treatment. Cell number was assayed using a colorimetric MTT assay. The optical density measurements were converted to cell number using a standard curve. The bars indicated represent standard deviation from the mean of triplicate samples.*

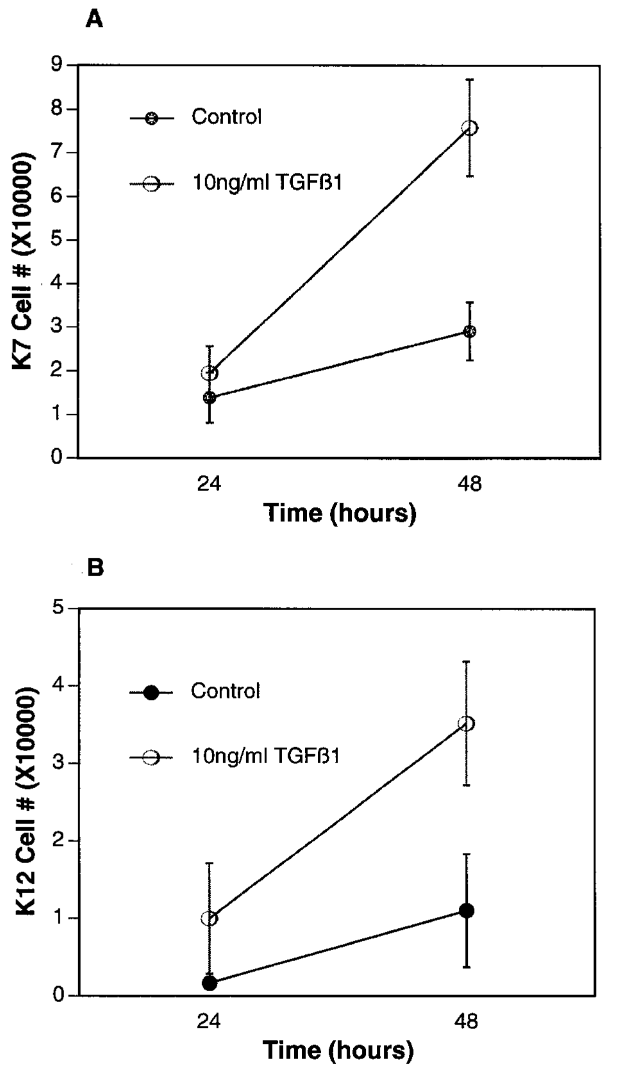

**Figure 4**. *Effect of exogenous TGF-β1 on K7 (A) and K12 (B). K7 and K12 cell lines were plated in media containing 10% FBS at a density of 5×10$^3$/well and 7.5×10$^3$/well, respectively. Twenty-four hours later, cells were incubated with 10 ng/ml of TGF-β1 in serum-free media. The control cells were treated with 4 mM HCl and 1 mg/ml BSA (vehicle for the exogenous TGF-β). Cells were harvested at 24 and 48 h after treatment. Cell number was assayed using MTT assay. The optical density measurements were converted to cell number using a standard curve. The bars indicated represent standard deviation from the mean of triplicate samples.*

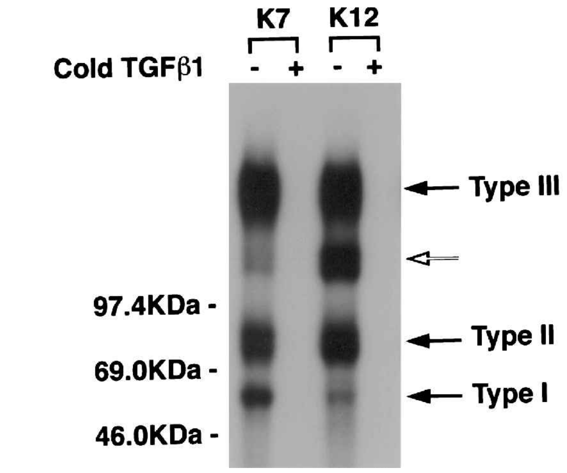

**Figure 5**. *TGF-β receptors in K7 and K12 cells. Osteosarcoma cells were incubated for 2.5 h with $^{125}$I labeled TGF-β1 with and without excess of unlabeled TGF-β1. Cells were lysed after a 1 h incubation with cross-linker. The proteins were separated on 8% Tris-Glycine polyacrylamide gels, dried and exposed to Kodak film. Each of the receptors is designated with a thin black arrow. An unexpected band migrating between the type II and type III receptor is noted with an open arrow.*

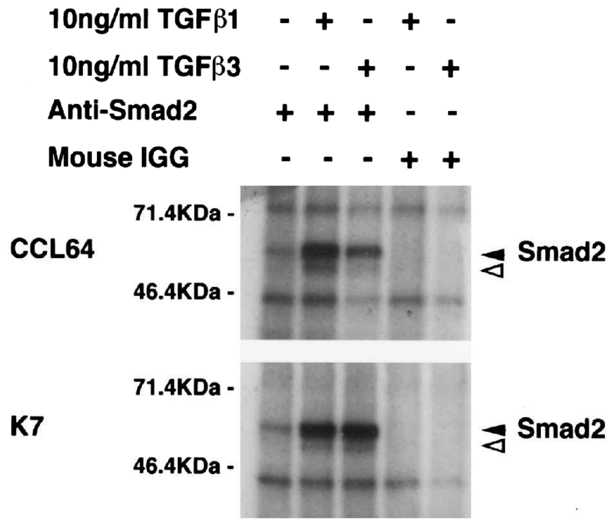

**Figure 6**. *Phosphorylation of Smad2 in CCL64 and K7 cells. Designated cells were metabolically labeled with [$^{32}$P] orthophosphate, treated for 30 minutes with 10 ng/ml of TGF-β1 or TGF-β3, and then immunoprecipitated with anti-Smad2 or mouse IgG. Precipitates were analyzed on a 6% Tris-Glycine polyacrylamide gel, dried and exposed to Kodak film. Phosphorylated Smad2 is indicated with a closed arrow. The smaller band indicated with the open arrow probably represents Smad3. Similar results were obtained for K12 cells (data not shown).*

show that the major proximal elements in the TGF-β signaling pathway are intact, namely the TGF-β receptors and Smads 2, 3 and 4. However, while ligand-induced phosphorylation of Smads 2 and 3 is associated with a reduction of phosphorylated pRb and growth arrest in mink lung epithelial cells (CCL64), culture of either K7 or K12 in the presence of either exogenous TGF-β or antibody to TGF-β has no effect on pRb phosphorylation status. The data suggest that the resistance to the growth inhibitory effect of TGF-β in these osteosarcoma cell lines results from the inability of normal receptor-initiated events to affect the phosphorylation of pRb. However, since TGF-β treatment did not alter the phosphorylation status of pRb, the mechanism for the growth stimulatory effects of TGF-β on these cells remains unclear.

The retinoblastoma protein has been established as an important mediator of growth inhibition in response to TGF-β. Herrera *et al.* showed that under certain growth conditions fibroblasts derived from RB-deficient mouse embryos (RB–/–) had a stimulatory growth response to TGF-β.[51] In addition, abnormalities in the retinoblastoma gene are found in greater than 50% of osteosarcomas, and are believed to play a role in the pathogenesis of this tumor.[52,53] Alterations in the pathway controlling

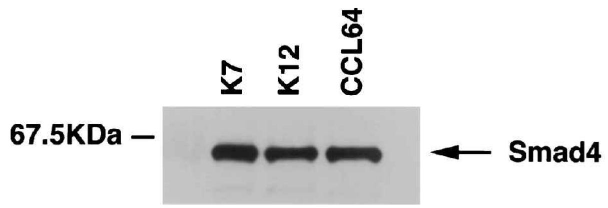

**Figure 7**. *Immunoblot detection of Smad4 in K7, K12 and CCL64 cells; 40 µg of protein from the designated sample was run on an 8% Tris-Glycine polyacrylamide gel, transferred overnight onto a nitrocellulose membrane and probed with mouse monoclonal antibody to Smad4.*

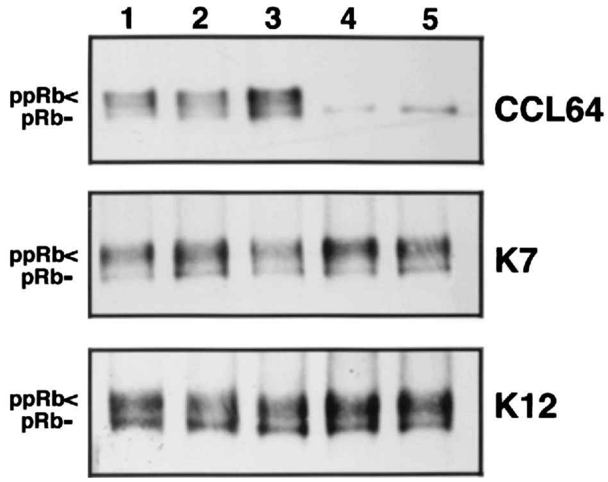

**Figure 8**. *Effect of treatment of cells with TGF-β1, TGFβ-3 and anti-TGF-β on phosphorylation of retinoblastoma protein. Cells were growth-arrested in serum-free media for 24 h prior to the addition of 5% FBS and 24 h treatment with either MOPC-21 (lane 1), 5 mg/ml anti-TGF-β (lane 2), HB buffer (solvent for TGF-β) (lane 3), 10 ng/ml TGF-β1 (lane 4) or 10 ng/ml TGF-β3 (lane 5); 40 mg of protein from the designated cell lines was electrophoresed on a 6% Tris-Glycine polyacrylamide gel, transferred onto a nitrocellulose membrane and probed with mouse anti-human Rb monoclonal antibody.*

phosphorylation of pRb may account for the lack of growth inhibitory response to TGF-β observed in our cell lines and would be functionally analogous to mutations found in human osteosarcomas. In the human osteosarcomas where there is no mutation in pRb detected one has to wonder if there are perturbations in the Rb pathway. Our mouse model for osteosarcoma allows one to study this possibility since pRb is intact but seems resistant to the growth inhibitory effects of TGF-β.

Active pRb (underphosphorylated form) acts to maintain cells in the G1 phase of the cell cycle. The events in the G1 phase of the cell cycle leading up to phosphorylation of pRb involve modulation of both the activity and the levels of cyclins and cdks as well as the cdk inhibitors. Aberrations in any of these components can potentially affect the cellular response to TGF-β. It has been shown that overexpression of cyclin E[54] or cyclin D[30] can overcome Rb-mediated growth arrest. Mutations in the INK family of cdk inhibitors, p15 and p16, have been described in a variety of malignancies including osteosarcoma.[55] p15 and p16 specifically bind and inhibit the activity of cdk4 and cdk6, which are key elements in phosphorylation of pRb. We are currently attempting to determine whether there is any disruption of the downstream effectors of the growth arrest response to TGF-β in K7 and K12 cells, particularly components of the cell cycle machinery involved in regulating pRb activity.

The lack of an effect of TGF-β on phosphorylation of Rb might also be a normal event in the physiologic response of cells of mesenchymal origin to TGF-β. Although a great deal is known about the pathways by which cells are growth-inhibited by TGF-β, there is little known about growth stimulatory pathways in normal cells. In osteoblasts derived from rat calvaria and fetal bovine bone cells, i.e. normal osteoblasts, both mitogenic and non-mitogenic effects of TGF-β have been reported.[4,10,56,57] These conflicting reports have been attributed to a specific inherent property of osteoblasts used in each study, such as their state of differentiation, or to differences in the cell populations and assay conditions. Based on these observations, we cannot discount the possibility that the growth stimulatory effect of TGF-β is a normal physiologic response to this cytokine for osteoblasts at a particular stage of differentiation. This would seem quite plausible since TGF-β is closely related to the bone morphogenic proteins which promote growth and differentiation in bone.

The disruption of TGF-β pathway in cancer has been demonstrated at the level of Smad signal transduction. Inactivating mutations and deletions have been found in Smad2 and Smad4 genes in a number of human cancers.[32,33,58,59] The phosphorylation of Smad2 and probably Smad3 is increased in response to TGF-β1 and TGF-β3 in K7 and K12 in the same manner as in Mv1Lu, and Smad4 is also expressed by both cell lines. These results indicate that the early post-receptor events occur as expected following exposure to ligand, and that in the persistence of an inactive Rb they might result ultimately in the mitogenic response. The potential influence of inhibitory Smads, Smad6 and Smad7, has not yet been evaluated, but may play a role despite the normal appearance of phosphorylated forms of Smad2 and Smad3 following receptor activation.[60,61]

In summary, we report the mitogenic potential of autocrine TGF-β as produced by two clonal murine osteosarcoma cell lines, K7 and K12. Our analysis predicts that this murine syngeneic tumor model is an appropriate *in vivo* system for determining how the production of TGF-β by osteosarcoma contributes to its growth. Further study of post-receptor signaling events will aim to identify factors dissociating normal ligand-induced activation of Smads in osteosarcoma from inhibition of pRb phosphorylation.

### References

1 Centrella M, Horowitz M, Wozney J, McCarthy T. Transforming growth factor-β gene family members and bone. *Endocr Rev* 1994;15:27–39.

2 Pfeilschifter J, D'souza S, Mundy G. Effects of transforming growth factor-β on osteoblastic osteosarcoma cells. *Endocrinology* 1987;121:212–8.

3 Pirskanen A, Jääskeläinen T, Mäenpää P. Effects of transforming growth factor β1 on the regulation of osteocalcin synthesis in human MG-63 osteosarcoma cells. *J Bone and Miner Res* 1994;9:1635–42.

4 Robey PG, Young MF, Flanders KC, Roche NS, Kondaiah P, Reddi A *et al.* Osteoblasts synthesize and respond to transforming growth factor-type β (TGFβ) *in vitro*. *J Cell Biol* 1987;105:457–63.

5 Centrella M. Growth-factor receptors and responses: a comparison of normal bone and osteosarcoma derived cell cultures. In: Novak J, McMaster J, eds. *Frontiers of osteosarcoma research: interdisciplinary survey of clinical and research advances*. Seattle: Hogrefe & Huber, 1993:457–68.

6 Kloen P, Jennings C, Gebhardt M, Springfield D, Mankin H. Expression of transforming growth factor-beta (TGF-β) receptors, TGF-β1 and TGF-β2 production and autocrine growth control in osteosarcoma cells. *Int J Cancer* 1994;58:440–5.

7 Yang R-S, Wu C-T, Lin K-H, Hong RL, Liu T-K, Lin K-S. Relation between histological intensity of transforming growth factor-β isoforms in human osteosarcoma and the rate of lung metastasis. *Tohoku J Exp Med* 1998;184:133–42.

8 Franchi A, Arganini L, Baroni G, Calzolari A, Capanna R, Campanacci D *et al.* Expression of transforming growth factor β isoforms in osteosarcoma variants: association of TGFβ1 with high-grade osteosarcomas. *J Pathol* 1998;185:284–9.

9 Kloen P, Gebhardt M, Perez-Atayde A, Rosenberg A, Springfield D, Gold L *et al.* Expression of transforming growth factor-β (TGF-β) isoforms in osteosarcomas. *Cancer* 1997;80:2230–9.

10 ten Dijke P, Iwata KK, Goddard C, Pieler C, Canals E, McCarthy TL *et al.* Recombinant transforming growth factor type β3: biological activities and receptor-binding properties in isolated bone cells. *Mol and Cell Biol* 1990;10:4473–9.

11 Massagué J. The transforming growth factor-β family. *Ann Rev Cell Biol* 1990;6:597–641.

12 Massagué J. Receptors for the TGF-β family. *Cell* 1992;69:1067–70.

13 Graycar J, Miller D, Arrick B, Lyons R, Moses H, Derynck R. Human transforming growth factor-β3: recombinant expression, purification, and biological activities in comparison with transforming growth factors-β1 and -β2. *Mol Endocrinol* 1989;3:1977–86.

14 Wang X-F, Lin HY, Ng-Eaton E, Downward J, Lodish HF, Weinberg RA. Expression cloning and characterization of the TGF-β type III receptor. *Cell* 1991;67:797–805.

15 Lopez-Casillas F, Wrana JL, Massagué J. Betaglycan presents ligand to the TGF-β signaling receptor. *Cell* 1993;73:1435–44.

16 Wrana JL, Attisano L, Carcamo J, Zentella A, Doody J, Laiho M *et al.* TGFβ signals through a heteromeric protein kinase receptor complex. *Cell* 1992;71:1003–14.

17 Liu X, Sun Y, Constantinescu S, Karam E, Weinberg R, Lodish H. Transforming growth factor β-induced phosphorylation of Smad3 is required for growth inhibition and transcriptional induction in epithelial cells. *Proc Natl Acad Sci USA* 1997;94:10669–74.

18 Nakao A, Imamura T, Souchelnytskyi S, Kawabata M, Ishisaki A, Oeda E *et al.* TGF-β receptor-mediated signalling through Smad2, Smad3, and Smad4. *EMBO J* 1997;16:5353–62.

19 Nakao A, Ršijer E, Imamura T, Souchelnytskyi S, Stenman G, Heldin C-H *et al.* Identification of Smad2, a human Mad-related protein in the transforming growth factor b signaling pathway. *J Biol Chem* 1997;272:2896–900.

20 Derynck R, Zhang Y, Feng X-H. Smads: transcriptional activators of TGF-β responses. *Cell* 1998;95:737–40.

21 Lagna G, Hata A, Hemmati-Brivanlou A, Massagué J. Partnership between DPC4 and SMAD proteins in TGF-β signaling pathways. *Nature* 1996;383:832–6.

22 Yanagi Y, Suzawa M, Kawabata M, Miyazono K, Yanagisawa J, Kato S. Positive and negative modulation of vitamin D receptor function by transforming growth factor-beta signaling through Smad proteins. *J Biol Chem* 1999;274:12971–4.

23 Yanagisawa J, Yanagi Y, Masuhiro Y, Suzawa M, Watanabe M, Kashiwagi K *et al.* Convergence of transforming growth factor-β and vitamin D signaling pathways on SMAD transcriptional coactivators. *Science* 1999;283:1317–21.

24 Alexandrow MG, Moses HL. Transforming growth factor β and cell cycle regulation. *Cancer Res* 1995;55:1452–7.

25 Ewen ME, Sluss HK, Whitehouse LL, Livingston DM. TGFb inhibition of cdk4 synthesis is linked to cell cycle arrest. *Cell* 1993;74:1009–20.

26 Hannon G, Beach D. p15INK4B is a potential effector of TGF-β-induced cell cycle arrest. *Nature* 1994;371:257–61.

27 Reynisdottir I, Polyak K, Iavarone A, Massagué J. Kip/Cip and Ink4 cdk inhibitors cooperate to induce cell cycle arrest in response to TGF-β. *Genes & Dev* 1995;9:1831–45.

28 Polyak K, Kato J, Solomon M, Sherr C, Massagué J, Roberts J *et al.* p27Kip1, a cyclin-cdk inhibitor, links transforming growth factor-β and contact inhibition to cell cycle arrest. *Genes & Dev* 1994;8:9–22.

29 Laiho M, DeCaprio JA, Ludlow JW, Livingston DM, Massagué J. Growth inhibition by TGF-β linked to suppression of retinoblastoma protein phosphorylation. *Cell* 1990;62:175–85.

30 Dowdy S, Hinds P, Louie K, Reed S, Arnold A, Weinberg R. Physical interaction of the retinoblastoma protein with human D cyclins. *Cell* 1993;73:499–511.

31 Weinberg R. The retinoblastoma protein and cell cycle control. *Cell* 1995;81:323–30.

32 Eppert K, Scherer S, Ozcelik H, Pirone R, Hoodless P, Kim H *et al.* MADR2 maps to 18q21 and encodes a TGFβ-regulated MAD-related protein that is functionally mutated in colorectal carcinoma. *Cell* 1996;86:543–52.

33 Nagatake M, Takagi Y, Osada H, Uchida K, Mitsudomi T, Saji S *et al.* Somatic *in vivo* alterations of DPC4 gene at 18q21 in human lung cancer. *Cancer Res* 1996;56:2718–20.

34 Markowitz SD, Roberts AB. Tumor suppressor activity of the TGF-β pathway in human cancers. *Cytokine & Growth Factor Rev* 1996;7:93–102.

35 Kimchi A, Wang X-F, Weinberg RA, Cheifetz S, Massagué J. Absence of TGF-β receptors and growth inhibitory responses in retinoblastoma cells. *Science* 1988;240:196–9.

36 Sun L, Wu G, Willson JK, Zborowska E, Yang J, Rajkarunanayake I *et al.* Expression of transforming growth factor b type II receptor leads to reduced malignancy in human breast cancer MCF-7 cells. *J Biol Chem* 1994;269:26449–55.

37 Mooradian D, McCarthy J, Komanduri K, Furcht L. Effects of transforming growth factor-β1 on human pulmonary adenocarcinoma cell adhesion, motility, and invasion *in vitro*. *J Natl Cancer Inst* 1992;84:523–7.
38 Welch D, Fabra A, Nakajima M. Transforming growth factor b stimulates mammary adenocarcinoma cell invasion and metastatic potential. *Proc Natl Acad Sci USA* 1990;87:7678–82.
39 Taipale J, Saharinen J, Keski-Oja J. Extracellular matrix-associated transforming growth factor-β: role in cancer cell growth and invasion. *Adv in Cancer Res* 1998;75:87–134.
40 Hojo M, Morimoto T, Maluccio M, Asano T, Morimoto K, Lagman M *et al.* Cyclosporine induces cancer progression by a cell-autonomous mechanism. *Nature* 1999;397:530–4.
41 Bšttinger E, Jakubczak J, Robert I, Mumy M, Hemmati P, Bagnall K *et al.* Expression of a dominant-negative mutant TGF-β type II receptor in transgenic mice reveals essential role for TGF-β in regulation of growth and differentiation in the exocrine pancreas. *EMBO J* 1997;16:2621–33.
42 Tang B, Bšttinger E, Jakowlew SB, Bagnall KM, Mariano J, Anver M *et al.* Transforming growth factor-β1 is a new form of tumor suppressor with true haploid insufficiency. *Nat Med* 1998;4:802–7.
43 Schmidt J, Straub G, Schšn A, Luz A, Murray A, Melchiori A, Aresu O *et al.* Establishment and characterization of osteogenic cell lines from a spontaneous murine osteosarcoma. *Differentiation* 1988;39:151–60.
44 Danielpour D, Kim K, Dart L, Watanabe S, Roberts A, Sporn M. Sandwich enzyme-linked immunosorbent assays (SELISAs) quantitate and distinguish two forms of transforming growth factor-beta (TGF-β1 and TGF-β2) in complex biological fluids. *Growth Factors* 1989;2:61–71.
45 Flanders KC, Thompson NL, Cissel DS, Obberghen-Schilling EV, Baker CC, Kass MD *et al.* Transforming growth factor-β1: histochemical localization with antibodies to different epitopes. *J Cell Biol* 1989;108:653–60.
46 Flanders KC, Cissel DS, Mullen LT, Danielpour D, Sporn MB, Roberts AB. Antibodies to transforming growth factor-β2 peptides: specific detection of TGF-β2 in immunoassays. *Growth Factors* 1990;3:45–52.
47 Flanders KC, Lubecke G, Engels S, Cissel DS, Roberts AB, Kondaiah P *et al.* Localization and actions of transforming growth factor-βs in the embryonic nervous system. *Development* 1991;113:183–91.
48 Denizot F, Lang R. Rapid colorimetric assay for cell growth and survival. *J Immunol Methods* 1986;89:271–7.
49 El-Brady O, Romanus J, Helman L, Cooper M, Rechler M, Israel M. Autonomous growth of a human neuroblastoma cell line is mediated by insulin-like growth factor II. *J Clin Invest* 1989;84:829–39.
50 Bšttinger E, Factor VM, Tsang ML, Weatherbee JA, Kopp JB, Qian SW *et al.* The recombinant proregion of transforming growth factor β1 (latency-associated peptide) inhibits active transforming growth factor β1 in transgenic mice. *Proc Natl Acad Sci USA* 1996;93:5877–82.
51 Herrera RE, Mäkelä TP, Weinberg RA. TGFb-induced growth inhibition in primary fibroblasts requires the retinoblastoma protein. *Mol Biol Cell* 1996;7:1335–42.
52 Wadayama B, Toguchida J, Shimizu T, Ishizaki K, Sasaki M, Kotoura Y *et al.* Mutation spectrum of the retinoblastoma gene in osteosarcoma. *Cancer Res* 1994;54:3042–8.
53 Benassi M, Molendini L, Gamber G, Sollazzo M, Ragazzini P, Merli M *et al.* Altered G1 phase regulation in osteosarcoma. *Int J Cancer* 1997;74:518–22.
54 Hinds P, Mittnacht S, Dulic V, Arnold A, Reed S, Weinberg R. Regulation of retinoblastoma protein functions by ectopic expresssion of human cyclins. *Cell* 1992;70:993–1006.
55 Miller C, Aslo A, Campbell M, Kawamata N, Lampkin B, Koeffler H. Alterations of the p15, p16, and p18 genes in osteosarcoma. *Cancer Genet Cytogenet* 1996;86:136–42.
56 Centrella M, McCarthy TL, Canalis E. Transforming growth factor β is a bifunctional regulator of replication and collagen synthesis in osteoblast-enriched cell cultures from fetal rat bone. *J Biol Chem* 1987;262:2869–74.
57 Rosen DM, Stempien SA, Thompson AY, Seyedin SM. Transforming growth factor-beta modulates the expression of osteoblast and chondroblast phenotypes *in vitro*. *J Cell Physiol* 1988;134:337–46.
58 Hahn S, Schutte M, Hoque A, Moskaluk C, Costa LD, Rozenblum E *et al.* DPC4, a candidate tumor suppressor gene at human chromosome 18q21.1. *Science* 1996;271:350–3.
59 Schutte M, Hruban R, Hedrick L, Cho K, Nadasdy G, Weinstein C *et al.* DPC4 gene in various tumor types. *Cancer Res* 1996;56:2527–30.
60 Imamura T, Takase M, Nishihara A, Oeda E, Hanal J, Kawabata M *et al.* Smad6 inhibits signalling by the TGF-β superfamily. *Nature* 1997;389:622–6.
61 Nakao A, Afrakhte M, Moren A, Nakayama T, Christian JL, Heuchel R *et al.* Identification of Smad7, a TGFβ-inducible antagonist of TGF-β signalling. *Nature* 1997;389:631–5.

## References

[PDF_00749] Alexandrow M. G., Moses H. L. (1995). Transforming growth factor beta and cell cycle regulation.. Cancer Res.

[PDF_00866] Benassi M. S., Molendini L., Gamberi G., Sollazzo M. R., Ragazzini P., Merli M., Magagnoli G., Sangiorgi L., Bacchini P., Bertoni F. (1997). Altered G1 phase regulation in osteosarcoma.. Int J Cancer.

[PDF_00853] Böttinger E. P., Factor V. M., Tsang M. L., Weatherbee J. A., Kopp J. B., Qian S. W., Wakefield L. M., Roberts A. B., Thorgeirsson S. S., Sporn M. B. (1996). The recombinant proregion of transforming growth factor beta1 (latency-associated peptide) inhibits active transforming growth factor beta1 in transgenic mice.. Proc Natl Acad Sci U S A.

[PDF_00813] Böttinger E. P., Jakubczak J. L., Roberts I. S., Mumy M., Hemmati P., Bagnall K., Merlino G., Wakefield L. M. (1997). Expression of a dominant-negative mutant TGF-beta type II receptor in transgenic mice reveals essential roles for TGF-beta in regulation of growth and differentiation in the exocrine pancreas.. EMBO J.

[PDF_00657] Centrella M., Horowitz M. C., Wozney J. M., McCarthy T. L. (1994). Transforming growth factor-beta gene family members and bone.. Endocr Rev.

[PDF_00877] Centrella M., McCarthy T. L., Canalis E. (1987). Transforming growth factor beta is a bifunctional regulator of replication and collagen synthesis in osteoblast-enriched cell cultures from fetal rat bone.. J Biol Chem.

[PDF_00828] Danielpour D., Kim K. Y., Dart L. L., Watanabe S., Roberts A. B., Sporn M. B. (1989). Sandwich enzyme-linked immunosorbent assays (SELISAs) quantitate and distinguish two forms of transforming growth factor-beta (TGF-beta 1 and TGF-beta 2) in complex biological fluids.. Growth Factors.

[PDF_00847] Denizot F., Lang R. (1986). Rapid colorimetric assay for cell growth and survival. Modifications to the tetrazolium dye procedure giving improved sensitivity and reliability.. J Immunol Methods.

[PDF_00734] Derynck R., Zhang Y., Feng X. H. (1998). Smads: transcriptional activators of TGF-beta responses.. Cell.

[PDF_00770] Dowdy S. F., Hinds P. W., Louie K., Reed S. I., Arnold A., Weinberg R. A. (1993). Physical interaction of the retinoblastoma protein with human D cyclins.. Cell.

[PDF_00849] El-Badry O. M., Romanus J. A., Helman L. J., Cooper M. J., Rechler M. M., Israel M. A. (1989). Autonomous growth of a human neuroblastoma cell line is mediated by insulin-like growth factor II.. J Clin Invest.

[PDF_00775] Eppert K., Scherer S. W., Ozcelik H., Pirone R., Hoodless P., Kim H., Tsui L. C., Bapat B., Gallinger S., Andrulis I. L. (1996). MADR2 maps to 18q21 and encodes a TGFbeta-regulated MAD-related protein that is functionally mutated in colorectal carcinoma.. Cell.

[PDF_00752] Ewen M. E., Sluss H. K., Whitehouse L. L., Livingston D. M. (1993). TGF beta inhibition of Cdk4 synthesis is linked to cell cycle arrest.. Cell.

[PDF_00839] Flanders K. C., Cissel D. S., Mullen L. T., Danielpour D., Sporn M. B., Roberts A. B. (1990). Antibodies to transforming growth factor-beta 2 peptides: specific detection of TGF-beta 2 in immunoassays.. Growth Factors.

[PDF_00843] Flanders K. C., Lüdecke G., Engels S., Cissel D. S., Roberts A. B., Kondaiah P., Lafyatis R., Sporn M. B., Unsicker K. (1991). Localization and actions of transforming growth factor-beta s in the embryonic nervous system.. Development.

[PDF_00834] Flanders K. C., Thompson N. L., Cissel D. S., Van Obberghen-Schilling E., Baker C. C., Kass M. E., Ellingsworth L. R., Roberts A. B., Sporn M. B. (1989). Transforming growth factor-beta 1: histochemical localization with antibodies to different epitopes.. J Cell Biol.

[PDF_00687] Franchi A., Arganini L., Baroni G., Calzolari A., Capanna R., Campanacci D., Caldora P., Masi L., Brandi M. L., Zampi G. (1998). Expression of transforming growth factor beta isoforms in osteosarcoma variants: association of TGF beta 1 with high-grade osteosarcomas.. J Pathol.

[PDF_00705] Graycar J. L., Miller D. A., Arrick B. A., Lyons R. M., Moses H. L., Derynck R. (1989). Human transforming growth factor-beta 3: recombinant expression, purification, and biological activities in comparison with transforming growth factors-beta 1 and -beta 2.. Mol Endocrinol.

[PDF_00886] Hahn S. A., Schutte M., Hoque A. T., Moskaluk C. A., da Costa L. T., Rozenblum E., Weinstein C. L., Fischer A., Yeo C. J., Hruban R. H. (1996). DPC4, a candidate tumor suppressor gene at human chromosome 18q21.1.. Science.

[PDF_00755] Hannon G. J., Beach D. (1994). p15INK4B is a potential effector of TGF-beta-induced cell cycle arrest.. Nature.

[PDF_00859] Herrera R. E., Mäkelä T. P., Weinberg R. A. (1996). TGF beta-induced growth inhibition in primary fibroblasts requires the retinoblastoma protein.. Mol Biol Cell.

[PDF_00869] Hinds P. W., Mittnacht S., Dulic V., Arnold A., Reed S. I., Weinberg R. A. (1992). Regulation of retinoblastoma protein functions by ectopic expression of human cyclins.. Cell.

[PDF_00809] Hojo M., Morimoto T., Maluccio M., Asano T., Morimoto K., Lagman M., Shimbo T., Suthanthiran M. (1999). Cyclosporine induces cancer progression by a cell-autonomous mechanism.. Nature.

[PDF_00893] Imamura T., Takase M., Nishihara A., Oeda E., Hanai J., Kawabata M., Miyazono K. (1997). Smad6 inhibits signalling by the TGF-beta superfamily.. Nature.

[PDF_00787] Kimchi A., Wang X. F., Weinberg R. A., Cheifetz S., Massagué J. (1988). Absence of TGF-beta receptors and growth inhibitory responses in retinoblastoma cells.. Science.

[PDF_00692] Kloen P., Gebhardt M. C., Perez-Atayde A., Rosenberg A. E., Springfield D. S., Gold L. I., Mankin H. J. (1997). Expression of transforming growth factor-beta (TGF-beta) isoforms in osteosarcomas: TGF-beta3 is related to disease progression.. Cancer.

[PDF_00677] Kloen P., Jennings C. L., Gebhardt M. C., Springfield D. S., Mankin H. J. (1994). Expression of transforming growth factor-beta (TGF-beta) receptors, TGF-beta 1 and TGF-beta 2 production and autocrine growth control in osteosarcoma cells.. Int J Cancer.

[PDF_00736] Lagna G., Hata A., Hemmati-Brivanlou A., Massagué J. (1996). Partnership between DPC4 and SMAD proteins in TGF-beta signalling pathways.. Nature.

[PDF_00766] Laiho M., DeCaprio J. A., Ludlow J. W., Livingston D. M., Massagué J. (1990). Growth inhibition by TGF-beta linked to suppression of retinoblastoma protein phosphorylation.. Cell.

[PDF_00720] Liu X., Sun Y., Constantinescu S. N., Karam E., Weinberg R. A., Lodish H. F. (1997). Transforming growth factor beta-induced phosphorylation of Smad3 is required for growth inhibition and transcriptional induction in epithelial cells.. Proc Natl Acad Sci U S A.

[PDF_00714] López-Casillas F., Wrana J. L., Massagué J. (1993). Betaglycan presents ligand to the TGF beta signaling receptor.. Cell.

[PDF_00784] Markowitz S. D., Roberts A. B. (1996). Tumor suppressor activity of the TGF-beta pathway in human cancers.. Cytokine Growth Factor Rev.

[PDF_00703] Massagué J. (1992). Receptors for the TGF-beta family.. Cell.

[PDF_00701] Massagué J. (1990). The transforming growth factor-beta family.. Annu Rev Cell Biol.

[PDF_00873] Miller C. W., Aslo A., Campbell M. J., Kawamata N., Lampkin B. C., Koeffler H. P. (1996). Alterations of the p15, p16,and p18 genes in osteosarcoma.. Cancer Genet Cytogenet.

[PDF_00796] Mooradian D. L., McCarthy J. B., Komanduri K. V., Furcht L. T. (1992). Effects of transforming growth factor-beta 1 on human pulmonary adenocarcinoma cell adhesion, motility, and invasion in vitro.. J Natl Cancer Inst.

[PDF_00780] Nagatake M., Takagi Y., Osada H., Uchida K., Mitsudomi T., Saji S., Shimokata K., Takahashi T., Takahashi T. (1996). Somatic in vivo alterations of the DPC4 gene at 18q21 in human lung cancers.. Cancer Res.

[PDF_00896] Nakao A., Afrakhte M., Morén A., Nakayama T., Christian J. L., Heuchel R., Itoh S., Kawabata M., Heldin N. E., Heldin C. H. (1997). Identification of Smad7, a TGFbeta-inducible antagonist of TGF-beta signalling.. Nature.

[PDF_00725] Nakao A., Imamura T., Souchelnytskyi S., Kawabata M., Ishisaki A., Oeda E., Tamaki K., Hanai J., Heldin C. H., Miyazono K. (1997). TGF-beta receptor-mediated signalling through Smad2, Smad3 and Smad4.. EMBO J.

[PDF_00729] Nakao A., Röijer E., Imamura T., Souchelnytskyi S., Stenman G., Heldin C. H., ten Dijke P. (1997). Identification of Smad2, a human Mad-related protein in the transforming growth factor beta signaling pathway.. J Biol Chem.

[PDF_00660] Pfeilschifter J., D'Souza S. M., Mundy G. R. (1987). Effects of transforming growth factor-beta on osteoblastic osteosarcoma cells.. Endocrinology.

[PDF_00762] Polyak K., Kato J. Y., Solomon M. J., Sherr C. J., Massague J., Roberts J. M., Koff A. (1994). p27Kip1, a cyclin-Cdk inhibitor, links transforming growth factor-beta and contact inhibition to cell cycle arrest.. Genes Dev.

[PDF_00758] Reynisdóttir I., Polyak K., Iavarone A., Massagué J. (1995). Kip/Cip and Ink4 Cdk inhibitors cooperate to induce cell cycle arrest in response to TGF-beta.. Genes Dev.

[PDF_00667] Robey P. G., Young M. F., Flanders K. C., Roche N. S., Kondaiah P., Reddi A. H., Termine J. D., Sporn M. B., Roberts A. B. (1987). Osteoblasts synthesize and respond to transforming growth factor-type beta (TGF-beta) in vitro.. J Cell Biol.

[PDF_00882] Rosen D. M., Stempien S. A., Thompson A. Y., Seyedin S. M. (1988). Transforming growth factor-beta modulates the expression of osteoblast and chondroblast phenotypes in vitro.. J Cell Physiol.

[PDF_00823] Schmidt J., Strauss G. P., Schön A., Luz A., Murray A. B., Melchiori A., Aresu O., Erfle V. (1988). Establishment and characterization of osteogenic cell lines from a spontaneous murine osteosarcoma.. Differentiation.

[PDF_00890] Schutte M., Hruban R. H., Hedrick L., Cho K. R., Nadasdy G. M., Weinstein C. L., Bova G. S., Isaacs W. B., Cairns P., Nawroz H. (1996). DPC4 gene in various tumor types.. Cancer Res.

[PDF_00791] Sun L., Wu G., Willson J. K., Zborowska E., Yang J., Rajkarunanayake I., Wang J., Gentry L. E., Wang X. F., Brattain M. G. (1994). Expression of transforming growth factor beta type II receptor leads to reduced malignancy in human breast cancer MCF-7 cells.. J Biol Chem.

[PDF_00805] Taipale J., Saharinen J., Keski-Oja J. (1998). Extracellular matrix-associated transforming growth factor-beta: role in cancer cell growth and invasion.. Adv Cancer Res.

[PDF_00819] Tang B., Böttinger E. P., Jakowlew S. B., Bagnall K. M., Mariano J., Anver M. R., Letterio J. J., Wakefield L. M. (1998). Transforming growth factor-beta1 is a new form of tumor suppressor with true haploid insufficiency.. Nat Med.

[PDF_00862] Wadayama B., Toguchida J., Shimizu T., Ishizaki K., Sasaki M. S., Kotoura Y., Yamamuro T. (1994). Mutation spectrum of the retinoblastoma gene in osteosarcomas.. Cancer Res.

[PDF_00710] Wang X. F., Lin H. Y., Ng-Eaton E., Downward J., Lodish H. F., Weinberg R. A. (1991). Expression cloning and characterization of the TGF-beta type III receptor.. Cell.

[PDF_00773] Weinberg R. A. (1995). The retinoblastoma protein and cell cycle control.. Cell.

[PDF_00801] Welch D. R., Fabra A., Nakajima M. (1990). Transforming growth factor beta stimulates mammary adenocarcinoma cell invasion and metastatic potential.. Proc Natl Acad Sci U S A.

[PDF_00717] Wrana J. L., Attisano L., Cárcamo J., Zentella A., Doody J., Laiho M., Wang X. F., Massagué J. (1992). TGF beta signals through a heteromeric protein kinase receptor complex.. Cell.

[PDF_00739] Yanagi Y., Suzawa M., Kawabata M., Miyazono K., Yanagisawa J., Kato S. (1999). Positive and negative modulation of vitamin D receptor function by transforming growth factor-beta signaling through smad proteins.. J Biol Chem.

[PDF_00744] Yanagisawa J., Yanagi Y., Masuhiro Y., Suzawa M., Watanabe M., Kashiwagi K., Toriyabe T., Kawabata M., Miyazono K., Kato S. (1999). Convergence of transforming growth factor-beta and vitamin D signaling pathways on SMAD transcriptional coactivators.. Science.

[PDF_00682] Yang R. S., Wu C. T., Lin K. H., Hong R. L., Liu T. K., Lin K. S. (1998). Relation between histological intensity of transforming growth factor-beta isoforms in human osteosarcoma and the rate of lung metastasis.. Tohoku J Exp Med.

[PDF_00696] ten Dijke P., Iwata K. K., Goddard C., Pieler C., Canalis E., McCarthy T. L., Centrella M. (1990). Recombinant transforming growth factor type beta 3: biological activities and receptor-binding properties in isolated bone cells.. Mol Cell Biol.

